# In Vitro Investigation of the Effects of Octenidine Dihydrochloride on Nasal Septum Squamous Carcinoma Cells

**DOI:** 10.3390/biomedicines13112668

**Published:** 2025-10-30

**Authors:** Ihsan Hakki Ciftci, Asuman Deveci Ozkan, Gulay Erman, Elmas Pinar Kahraman Kilbas, Mehmet Koroglu

**Affiliations:** 1 Department of Medical Microbiology, Faculty of Medicine, Sakarya University, Sakarya 54050, Türkiye; 2 Department of Medical Biology, Faculty of Medicine, Sakarya University, Sakarya 54050, Türkiye; 3 Health Services Education Research and Application Centre, Sakarya University, Sakarya 54050, Türkiye; 4 Department of Medical Biochemistry, Institute of Health Science, Sakarya University, Sakarya 54050, Türkiye; 5 Department of Medical Laboratory Techniques, Health Services Vocational School, Fenerbahce University, Istanbul 34758, Türkiye

**Keywords:** octenidine dihydrochloride, nasal mucosal cells, cytotoxicity, genotoxicity

## Abstract

**Background/Objectives**: The aim of this study was to investigate the cytotoxic, genotoxic, apoptotic, and anti-inflammatory effects of the antiseptic agent octenidine dihydrochloride (OCT-D) on the RPMI-2650 cell line derived from human nasal mucosa in vitro. **Methods**: RPMI-2650 cells and Human Umbilical Cord Endothelial Cells (HUVECs) were treated with various concentrations of OCT-D (0.00625–0.4%) for 12 and 24 h. Cell viability was assessed using the WST-1 assay, while DNA damage was assessed using the comet and micronucleus (MN) assays. Apoptotic activity was determined using Annexin V flow cytometry and fluorescence microscopy. Intracellular reactive oxygen species (ROS) levels were measured, and inflammatory cytokines (IL-1β, IL-6, TNF-α, and IFN-γ) were measured by Enzyme-Linked Immunosorbent Assay (ELISA). The mRNA expression of genes associated with apoptosis, oxidative stress, and inflammation was analyzed using RT-PCR. **Results**: OCT-D caused dose- and time-dependent cytotoxicity, and RPMI-2650 cells showed greater resistance compared to HUVECs. While a strong apoptotic response was observed in HUVECs, RPMI-2650 cells exhibited limited apoptosis. OCT-D was found to cause dose-dependent DNA damage and an increase in MN in both cell lines. OCT-D significantly reduced cytokine levels and ROS production in both cell types. RT-PCR results supported its anti-inflammatory and antioxidant effects at the molecular level. **Conclusions**: In conclusion, this study demonstrated that OCT-D exhibited minimal cytotoxic and apoptotic effects in RPMI-2650 cells, but affected vascular structure by inducing apoptosis in endothelial cells. These findings provide important evidence that OCT-D can be used as a potential adjunctive agent in nasal treatments, and these data need to be supported by preclinical and clinical studies.

## 1. Introduction

Octenidine dihydrochloride (OCT-D) is a topical antiseptic with broad-spectrum activity against Gram-positive and Gram-negative bacteria, fungi and some viruses. OCT-D is reportedly used as an approved antiseptic in combination with aliphatic alcohols (phenoxyethanol) in many European countries [1]. The antimicrobial activity of OCT-D demonstrates remarkable potential, particularly against multidrug-resistant (MDR) microorganisms. OCT-D has been shown to be highly effective even against multidrug-resistant Gram-negative pathogens in a very short time. It was reported to be effective for all isolates using different OCT-D concentrations (0.01% and 0.05%) with short contact times, and a bacterial reduction factor of >5 log_10_ was systematically observed [2].

MDR microorganisms pose a serious challenge to healthcare systems. Treatment of infections caused by these microorganisms is complex and costly because only second-line antibiotics are available [3]. Patients colonized with MDR microorganisms are at high risk of developing bacterial infection because they suffer from underlying comorbidities. These patients also represent a potential source of transmission, requiring specialized infection control measures to prevent further spread of MDR [4,5]. The use of OCT-D as a topical antiseptic may be effective in reducing the colonization and spread of MDR bacteria. This effect occurs because OCT-D binds to the bacterial cell membrane, penetrates the lipopolysaccharide layer, and disrupts membrane integrity via hydrocarbon chains, resulting in rapid lysis of the bacteria [6].

In this context, the elimination of antibiotic-resistant microorganisms, such as methicillin-resistant *Staphylococcus aureus* (MRSA), which can colonize the noses of hospital personnel, is of critical importance. The study by Mert et al. found nasal *S. aureus* carriage rates of 19.6%, 28.8%, and 31.8% among nurses, doctors, and allied health personnel, respectively, highlights the seriousness of this issue [7]. Mupirocin resistance is generally associated with the presence of the mupA and mupB genes, which encode alternative forms of isoleucyl-tRNA synthetase, allowing the bacteria to evade the effects of mupirocin [8]. Chlorhexidine resistance is linked to genes such as qacA/qacB, which encode efflux pumps that expel chlorhexidine from the cell [9]. These resistance mechanisms can be overcome by the membrane-disrupting effects of OCT-D, as OCT-D targets the bacterial cell membrane in a non-specific manner, remaining effective independently of these genetic resistance mechanisms. The use of agents such as OCT-D for MDR microorganisms has become even more compelling due to the global increase in resistance to the topical antibiotic mupirocin and the questionable efficacy of chlorhexidine-based formulations [3]. However, our study is limited by in vitro data without microbiological assays, which should be addressed in future research.

However, although OCT-D has been tested as an antiseptic in animal mucosal tissues, it has not been adequately studied in vitro on human nasal mucosal cells, and the lack of cytotoxicity test data highlights the need for further research on this topic [10]. Additionally, in a study investigating the cytotoxic effects of OCT-D on oral epithelial cells, it was reported that OCT-D in mouthwash form showed lower toxic effects on both fibroblasts and epithelial cells compared to other antiseptics tested, such as chlorhexidine [11]. This creates a substantial knowledge gap regarding the evaluation of OCT-D’s safe and effective use for nasal antisepsis.

The nasal septum squamous carcinoma cell line (RPMI-2650) is a model consisting of squamous carcinoma cells obtained from the nasal septum of a 52-year-old male patient and exhibiting epithelial morphology [12]. This cell line is reported to be suitable for nasal drug studies because it yields positive results in drug permeation assays and provides superior discrimination between high-permeability model drugs and low-permeability category drugs [13]. RPMI-2650 cells are widely used in in vitro studies of nasal drug delivery due to their epithelial morphology and permeability properties. However, as cells of carcinoma origin, they may exhibit altered metabolism, proliferation, and drug response compared to normal nasal epithelial cells, which should be considered when extrapolating results to healthy tissue.

This study aims to comprehensively investigate the cytotoxic, genotoxic, apoptotic, and anti-inflammatory effects of OCT-D on RPMI-2650 nasal septum squamous carcinoma cells in vitro.

## 2. Materials and Methods

### 2.1. Cell Culture Conditions

In our study, we used the RPMI-2650 and Human Umbilical Vein Endothelial Cells (HUVEC) as test groups. Cells were removed from the liquid nitrogen tank, thawed in a 37 °C water bath, and the freezing medium was removed. They were then seeded in appropriate media: Eagle’s Minimum Essential Medium (EMEM) supplemented with 8% Fetal Bovine Serum (FBS) and 1% penicillin for RPMI-2650; Dulbecco’s Modified Eagle’s Medium (DMEM) supplemented with 2 mM L-glutamine, 50 IU/mL penicillin, and 50 mg/mL streptomycin for HUVEC. Cells were expanded at 37 °C and 5% CO_2_ [14].

### 2.2. Viability Analysis

RPMI-2650 and HUVEC were seeded at 2 × 10^4^ cells/mL in 96-well microplates and incubated with different concentrations of OCT-D (0.4–0.00625%) for 12 to 24 h. Each condition was tested in triplicate wells (technical replicates) across three independent experiments (biological replicates). A volume of 10 µL of Water-Soluble Tetrazolium Salt-1 (WST-1) reagent was then added to each well and incubated at 37 °C for 1–4 h. Optical density was measured using an ELISA reader at a wavelength of 460–620 nm, and viability rates were calculated using untreated cells as baseline control.

### 2.3. Single Cell Gel Electrophoresis (Comet Assay)

To assess DNA damage, RPMI-2650 and HUVEC were seeded in 6-well plates at a density of 1 × 10^5^ cells/mL and treated with OCT-D. The treatment concentrations and exposure durations were selected based on the WST-1 assay results, which identify metabolically active, non-lethal doses rather than direct DNA damage. After treatment, analysis was performed using the Comet assay kit (Abcam, Cambridge, UK) according to the company’s recommended protocol. Cells were lysed for 1 h at 4 °C in a lysis buffer containing 2.5 M NaCl, 100 mM EDTA, 10 mM Tris, and 1% Triton X-100 (pH ≥ 10). DNA unwinding was performed in alkaline buffer (pH > 13) for 15 min at 4 °C. Electrophoresis was carried out at 0.7 V/cm (300 mA) for 20 min in an alkaline electrophoresis buffer (300 mM NaOH, 1 mM EDTA). Afterward, the slides were washed with Tris buffer and stained with Vista Green. Images were captured using a fluorescence microscope (Olympus BX53, Tokyo, Japan), and comet tail DNA percentages were quantified using the OpenComet plugin (ImageJ software, version 1.3, NIH, Bethesda, MD, USA). DNA percentage was calculated by examining 25 randomly selected nuclei under a fluorescence microscope [15].

### 2.4. Cytokinesis-Blocked Micronucleus (CBMN) Analysis

RPMI-2650 and HUVEC were seeded in 6-well culture plates at a density of 1 × 10^5^ cells/mL. For the micronucleus assay, cells were treated with OCT-D at concentrations selected based on WST-1 results to identify non-lethal, metabolically active doses. These doses were used as a reference for MN testing, acknowledging that they may not correspond precisely to genotoxic or clastogenic thresholds. Following treatment with WST-1 at the effective doses of OCT-D determined, the cells were added to medium containing 2 µg/mL cytochalasin-B and incubated for 24 h. After washing with PBS, the cells were fixed with 70% ethanol and stained with 5% Giemsa stain. Micronucleus counts were performed according to the Heddle and Countryman criteria, and the micronucleus index was assessed in at least 1000 binucleotic cells for each treatment [16].

### 2.5. Apoptosis and Reactive Oxygen Species (ROS) Flow Cytometry Analysis

To determine apoptotic activity and ROS levels, RPMI-2650 and HUVEC were seeded in 6-well plates at a density of 1 × 10^5^ cells/mL and treated with WST-1 at the most effective OCT-D dose and duration. Subsequently, for Annexin V analysis, cells were harvested with trypsin, washed with PBS, incubated with Annexin V dye for 30 min in the dark, and the percentage of apoptotic cells was analyzed by flow cytometry. Furthermore, cells were fixed with 4% paraformaldehyde, stained with acridine orange and DAPI, and morphological apoptotic changes were visually assessed under a fluorescence microscope [17]. For ROS analysis, cells were harvested with trypsin after OCT-D treatment, washed with PBS, and ROS-specific dyes were added and incubated in the dark for 30 min. Then, intracellular ROS amounts were analyzed by flow cytometry and oxidative stress levels were determined [18].

### 2.6. Enzyme-Linked Immunosorbent Assay (ELISA)

To determine the inflammatory effects of OCT-D, protein levels of IL-1β, IFN-γ, TNF-α, and IL-6 were measured in RPMI-2650 and HUVEC using appropriate ELISA kits. Supernatants collected from cell culture media were analyzed according to kit protocols, and cytokine amounts were calculated based on a standard curve [19]. Cytokine levels were normalized to the number of viable cells determined by WST-1 assay to account for differences in cell density and viability between treatment groups.

### 2.7. Real-Time Polymerase Chain Reaction (RT-PCR) Analysis

To investigate the apoptotic, DNA damage, and inflammatory effects of OCT-D at the molecular level, mRNA expression levels of *Bax*, *Bcl-2*, *caspase-3*, *ATM*, *BRCA1*, *Rad51*, *SOD*, *CAT*, *IL-1β*, *IFN-γ*, *TNF-α*, and *IL-6* genes were analyzed using RT-PCR. Total RNA was isolated from the cells, and cDNA was synthesized after RNA quality and quantity were checked with nanodrops. mRNA samples were collected at 3 h post-treatment for early-response apoptotic genes (*Bax*, *Bcl-2*, *caspase-3*) and at 24 h for DNA repair and antioxidant genes (*ATM*, *BRCA1*, *Rad51*, *SOD*, *CAT*), based on previous studies reporting peak expression times for these gene groups [20,21]. The prepared cDNA was amplified using an RT-PCR device using appropriate primers and a Syber Green probe [22].

### 2.8. Statistical Analysis

The obtained data were analyzed using SPSS software (IBM SPSS Statistics, Version 25.0; IBM Corp., Armonk, NY, USA) to assess significant differences between groups. Differences between multiple test groups were assessed using two-way ANOVA followed by Dunnett’s post hoc test for multiple comparisons. For pairwise comparisons, independent *t*-tests were used where appropriate. Data are presented as the mean ± standard deviation, and *p* < 0.05 was considered statistically significant. In the calculations, *p* < 0.05 was considered statistically significant.

## 3. Results

### 3.1. Effect of OCT-D on Cell Viability

**Figure 1:**
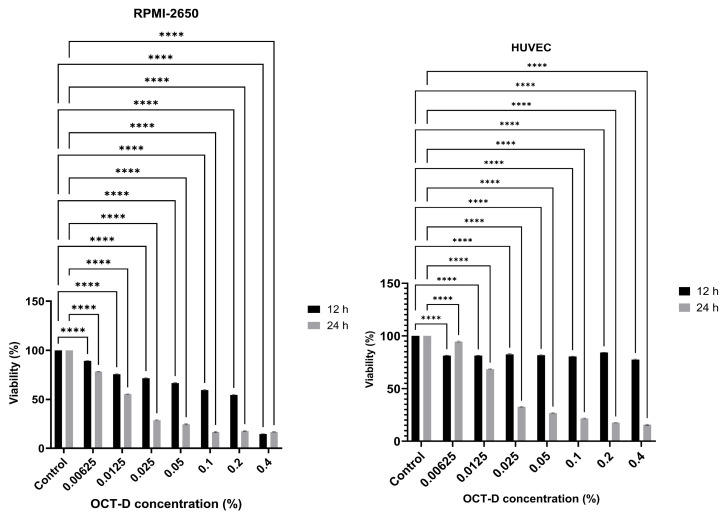
Evaluation of the cytotoxic effect of OCT-D on RPMI-2650 and HUVEC at 12 and 24 h (**** *p* < 0.0001).

After 12 h of application in RPMI-2650 cells, a progressive decrease in viability was observed with increasing concentration compared to the untreated cells. At the lowest concentration (0.00625%), viability remained above 89%, while at the 0.1% concentration, it decreased to approximately 59%. After 24 h of exposure, the cytotoxic effect became more pronounced, with viability decreasing to 16–17% at the 0.1% concentration (Figure 1).

HUVEC exhibited higher tolerance to OCT-D. After 12 h of application, viability was maintained at 81–82% at the 0.1% concentration, but decreased to 26–27% at 24 h. At the lowest concentrations (0.00625–0.0125%), HUVEC exhibited high viability (Figure 1). A dose-dependent cytotoxic effect was observed with increasing concentration in both cell lines, and a statistically significant difference was found between all concentrations (*p* < 0.05). The greater sensitivity of RPMI-2650 cells to OCT-D reflects the delicate nature of the nasal mucosa, while the high tolerance of HUVEC reflects the resistance of endothelial cells. The 0.1% concentration, which provides the optimal balance between antimicrobial efficacy and cellular safety, was determined as the IC_50_ value for both cell lines (Figure 1).

### 3.2. Effect of OCT-D on DNA Damage

**Figure 2:**
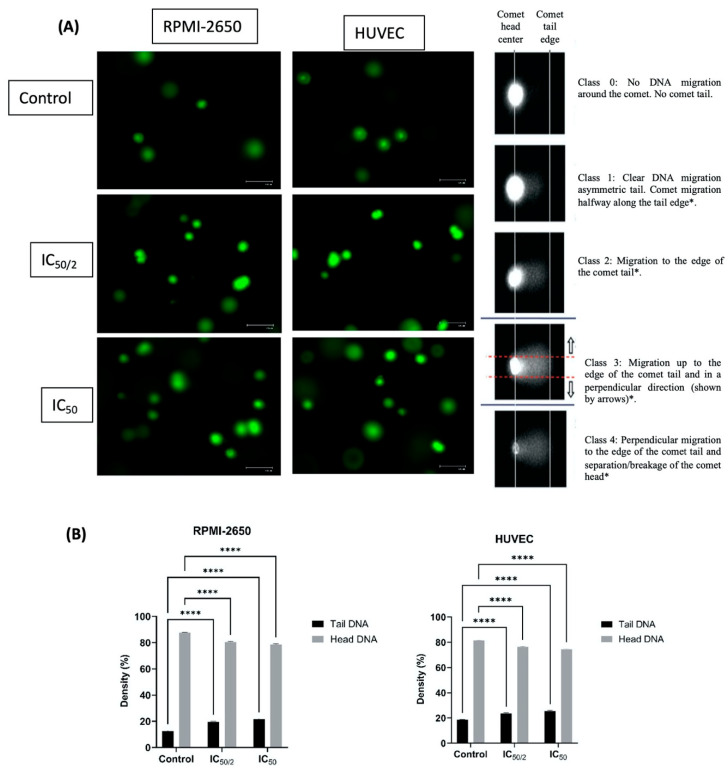
Evaluation and statistical analysis of the effect of OCT-D on DNA damage in RPMI-2650 and HUVEC by gel electrophoresis (SCGE, COMET) for 12 h (**A**). (**B**) [IC_50_: 50 percent inhibitory concentration (% 0.02 OCT-D), **** *p* < 0.0001]. The green fluorescent signal indicates DNA content. White arrows indicate cells with pronounced tail formation. The right panel illustrates typical classes used to grade DNA damage according to the Comet classification. * Modified from Møller et al., 2023 [23]. Scale bar = 50 µm.

In RPMI-2650 cells, the tail DNA percentage increased to 19.85% at the ½ IC_50_ concentration and to 21.29% at the IC_50_ concentration compared to untreated cells within the same cell line (12.25%). This increase demonstrated a dose-dependent genotoxic effect and was found to be statistically significant (*p* < 0.0001). No significant change was observed in the head DNA percentages compared to the untreated cells within the same cell line (Figure 2). The genotoxic effect was more pronounced in HUVEC. While the tail DNA percentage was 18.65% in the untreated cells, it increased to 23.91% and 25.14% at the ½ IC_50_ and IC_50_ concentrations, respectively. A slightly higher Tail DNA percentage in HUVEC controls may result from the inherent sensitivity of primary endothelial cells to handling-related oxidative and mechanical stress. All samples were processed under identical conditions; thus, this baseline variation does not affect the comparative interpretation of treatment-induced DNA damage. This increase was more pronounced than in RPMI-2650 cells, ndicating increased sensitivity of endothelial cells to OCT-D. The difference between both head DNA and tail DNA percentages was found to be statistically significant (*p* < 0.05). The lack of significant changes in head DNA percentages in both cell lines indicated that OCT-D primarily caused partial DNA damage and did not cause gross fragmentation (Figure 2). It should be noted that the comet assay is a semi-quantitative method and does not specifically distinguish double-strand breaks; therefore, head DNA reflects DNA retention rather than precise fragment size. This methodological limitation was taken into account when interpreting the results.

### 3.3. The Effect of OCT-D on MN Formation

**Figure 3:**
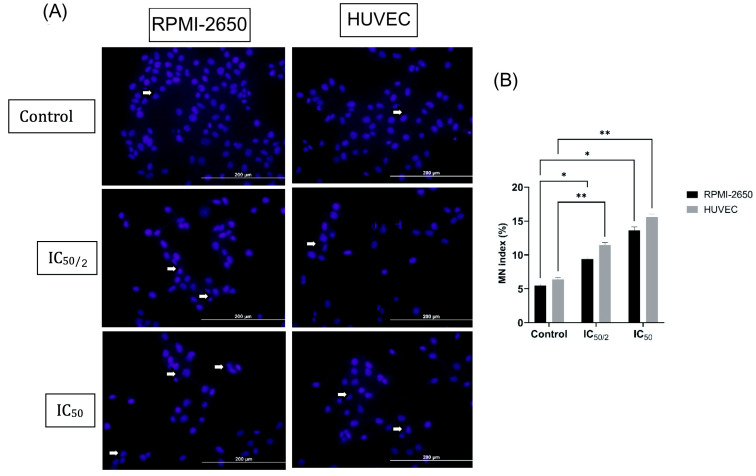
Evaluation of the effect of OCT-D on the amount of micronuclei (MN) in RPMI-2650 and HUVEC for 12 h. (**A**) Results of cytokinesis-blocked micronucleus (CBMN) analysis of the effect of OCT-D application on the amount of MN and (**B**) Statistical analysis [IC_50_: 50 percent inhibitory concentration (% 0.02 OCT-D), * *p* < 0.01, ** *p* < 0.001]. Blue fluorescent signal represents cell nuclei. White arrows indicate nuclei where MN formation was observed. Scale bar: 200 µm.

A dose-dependent increase in micronucleated MNs was observed in RPMI-2650 cells compared to untreated cells within the same cell line (5.2%) (Figure 3). The MN percentage increased to 9.12% at the ½ IC_50_ concentration and to 13.32% at the IC_50_ concentration. This increase was statistically significant (*p* < 0.01 for ½ IC_50_, *p* < 0.001 for ½ IC_50_) and demonstrated the genotoxic effect of OCT-D (Figure 3). A slightly higher baseline MN frequency was detected in test groups, likely reflecting culture-related background damage inherent to in vitro conditions. Nevertheless, all samples were treated and analyzed under identical conditions, allowing reliable comparison of treatment-induced MN formation.

The genotoxic effect was more pronounced in HUVEC. While the MN percentage in the untreated cells was 6.21%, it increased to 11.21% and 15.29% at the ½ IC_50_ and ½ IC_50_ concentrations, respectively. MN change in HUVEC was greater than in RPMI-2650 cells (*p* < 0.0001), indicating increased chromosomal damage sensitivity of endothelial cells to OCT-D (Figure 3).

### 3.4. Effect of OCT-D on Apoptotic Cell Percentage, Cell, and Nuclear Morphology

**Figure 4:**
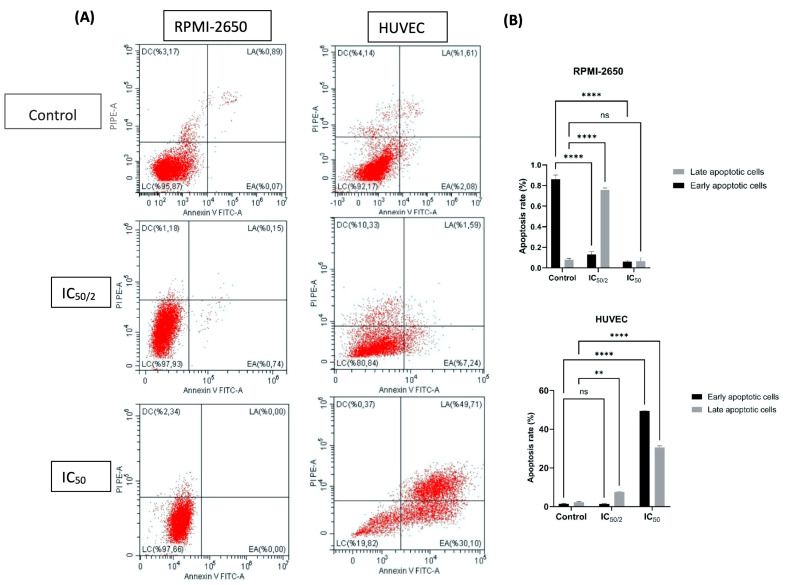
Effect of OCT-D on apoptosis in RPMI-2650 and HUVEC lines. (**A**) Following 24 h of OCT-D application in RPMI-2650 and HUVEC, apoptosis was analyzed by flow cytometry using Annexin V-FITC/PI staining. Representative dot plots for untreated and treated cells are shown. The lower left (LC) region shows live cells (Annexin V^−^/PI^−^), the lower right (EA) shows early apoptotic cells (Annexin V^+^/PI^−^), the upper right (LA) shows late apoptotic/necrotic cells (Annexin V^+^/PI^+^), and the upper left (DC) shows necrotic cells (Annexin V^−^/PI^+^). (**B**) The total apoptotic cell ratio (early + late apoptosis) in each group is summarized. Data are presented as the mean ± SD of three independent experiments (ns: not significant, ** *p* < 0.01, **** *p* < 0.0001).

Compared to untreated cells, no significant increase in apoptotic cell rates was observed with OCT-D application in RPMI-2650 cells (Figure 4). While the late apoptotic cell rate was 0.07% in the untreated cells, it was determined to be 0.74% and 0.6% at the ½ IC_50_ and IC_50_ doses, respectively (*p* < 0.0001). Early apoptosis rates remained similarly low (Figure 4).

In contrast, the apoptotic effect of OCT-D was significantly increased in HUVEC. The late apoptotic cell rate, which was 1.06% in the untreated cells, increased to 7.24% at the ½ IC_50_ dose and 30.01% at the IC_50_ dose (*p* < 0.01 and *p* < 0.0001). The total apoptotic cell rate reached 80%, demonstrating the high sensitivity of HUVEC to OCT-D (Figure 4). These results suggest that OCT-D triggers apoptotic cell death in endothelial cells and poses a potential risk for vascular toxicity. The relative apoptosis resistance of RPMI-2650 cells to OCT-D suggested that nasal epithelial cells exhibit a different response to cytotoxic effects. Furthermore, the high apoptotic response in HUVEC supported the sensitivity of endothelial cells to low-dose anticancer drugs and the role of apoptosis in the regression of peritumor neovascularization. Annexin V analysis, a sensitive and rapid method for detecting early apoptosis, provided reliable detection of cell-type-specific apoptotic responses.

**Figure 5:**
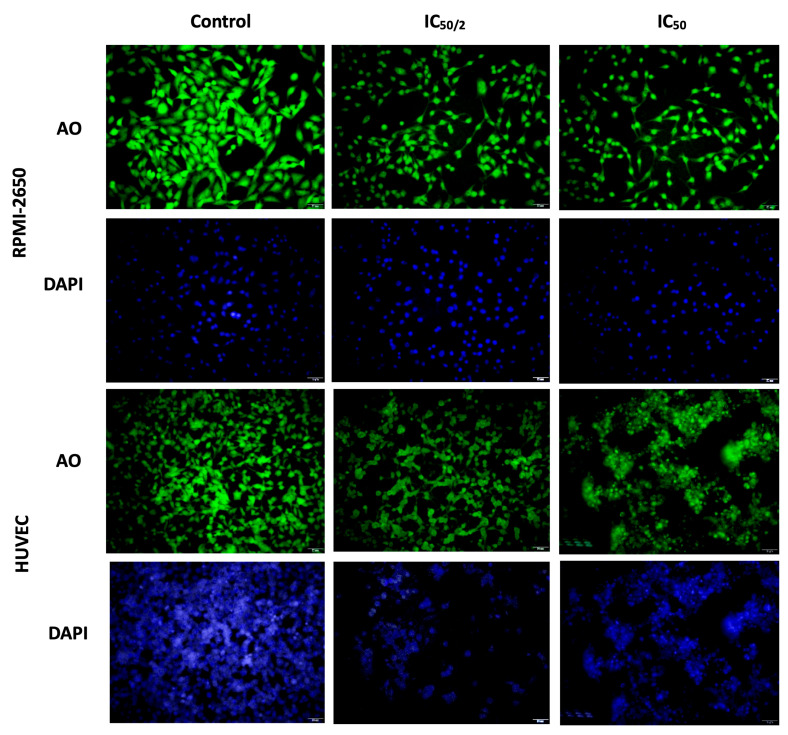
Evaluation of apoptosis and nuclear morphology in RPMI-2650 and HUVEC after OCT-D application by fluorescence microscopy. Cells were stained with AO (green) and DAPI (blue) after 12 h of OCT-D application. AO marked the cytoplasm/nucleus of viable and apoptotic cells, while DAPI stained the nuclei [IC_50_: 50% inhibitory concentration (0.05% OCT-D), AO: acridine orange]. Scale bar = 50 µm.

Fluorescence microscopy analyses using acridine orange (AO) and 4′,6-diamidino-2-phenylindole (DAPI) staining were performed to evaluate the apoptotic effects of OCT-D treatment on RPMI-2650 and HUVEC (Figure 5). Cells were treated with OCT-D at IC_50_ (0.1% OCT-D) and ½ IC_50_ (0.05% OCT-D) concentrations for 12 h. In these analyses, AO labeled the cytoplasm and nucleus of viable and apoptotic cells, while DAPI was used to specifically stain the nucleus. The images obtained revealed that morphological changes varied depending on the cell type and dose (Figure 5). No obvious morphological changes were observed in RPMI-2650 cells after OCT-D treatment; nuclear and cytoplasmic integrity were largely preserved. This indicated that this cell line was more resistant to OCT-D and less likely to induce apoptosis. In contrast, pronounced apoptotic morphological changes were observed in HUVEC, particularly at the IC_50_ dose. These changes included cell and nucleus shrinkage, plasma membrane blebbing, cytoplasmic condensation, and cell fragmentation (Figure 5). Cytoplasmic and nuclear morphology changes observed with AO and DAPI staining, consistent with Annexin V analyses, supported the occurrence of advanced stages of apoptosis in HUVEC. Nuclear blebbing, in particular, along with chromatin condensation and nuclear membrane disruption during apoptosis, were among the morphological indicators of cell death. Furthermore, it was determined that the spherical protrusions observed in the cell membrane developed due to cytoskeletal disruption and were part of the apoptotic progression process.

### 3.5. The Effect of OCT-D on ROS Quantification

**Figure 6:**
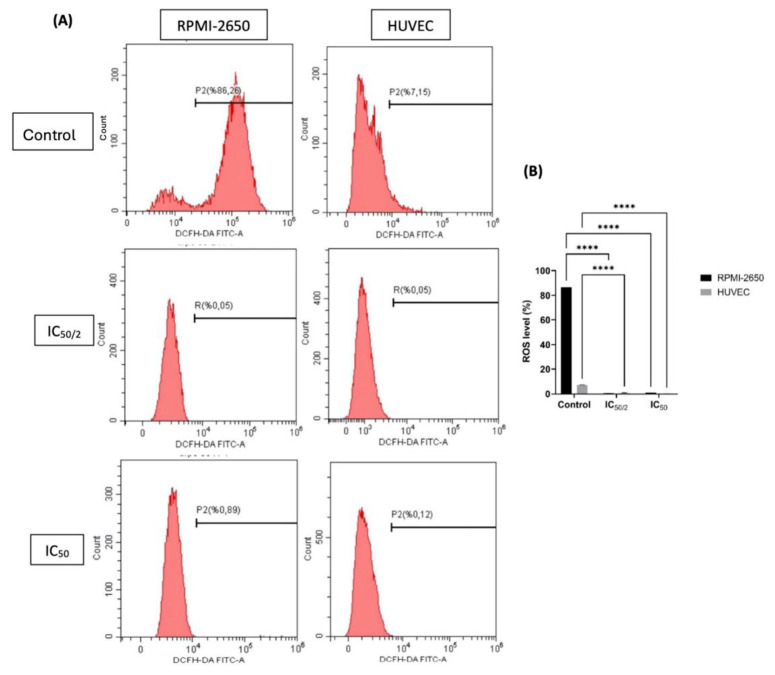
Analysis of reactive oxygen species (ROS) levels in RPMI-2650 and HUVEC after OCT-D application. (**A**) ROS production in cells was assessed by flow cytometry using the DCFH-DA fluorescent probe. Red histograms show the fluorescent signal from cell populations stained with DCFH-DA dye. (**B**) Total ROS ratios of each group were statistically evaluated. Data are given as the mean ± SD of three independent experiments [IC_50_: 50 percent inhibitory concentration (0.05% OCT-D)]. **** *p* < 0.0001.

Intracellular ROS levels were quantitatively measured in flow cytometry analyses using the DCFH-DA fluorescent probe (Figure 6). In RPMI-2650 cells, the average ROS level in the untreated cells was 88.15%, but after ½ IC_50_ and IC_50_ treatments, this rate decreased to 0.52% and 0.92%, respectively. This marked decrease suggested that OCT-D suppressed the oxidative stress response in these cells and exhibited a potential antioxidant effect (Figure 6). Similarly, in HUVEC, the average ROS level measured in the untreated cells was 7.26%, while in the ½ IC_50_ and IC_50_ groups, this rate decreased to 0.17% and 0.72%, respectively (Figure 6). The obtained data revealed that OCT-D treatment significantly reduced ROS production in both cell lines (*p* < 0.05).

### 3.6. Effect of OCT-D on Inflammatory Markers

**Figure 7:**
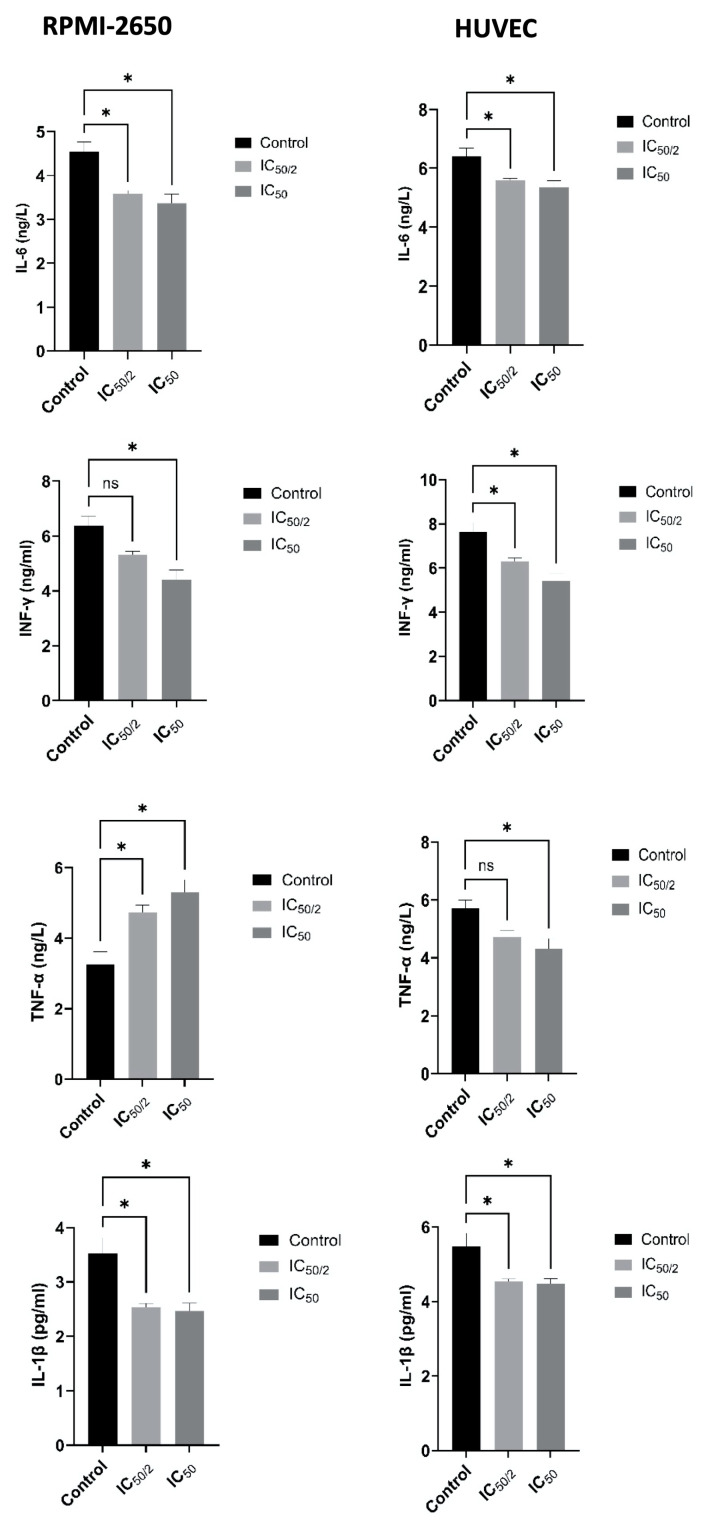
Calorimetric evaluation of the effect of OCT-D on the amount of IL-6, IL-1β, INF-γ and TNF-α in RPMI-50 and HUVEC for 12 h [IC_50_: 50 percent inhibitory concentration (% 0.02 OCT-D), ns: no significance, * *p* < 0.005)].

Calorimetric measurements performed after 12 h of application of OCT-D at IC_50_ (0.1%) and ½ IC_50_ (0.05%) concentrations revealed notable changes in cytokine levels in both cell lines (Figure 7). Analysis on RPMI-2650 cells revealed significant OCT-D-induced decreases in IL-6, IL-1β, and IFN-γ levels. While IL-1β levels were approximately 3.5 pg/mL in the untreated cells, this value decreased to 2.4–2.5 pg/mL in the IC_50_ treatment (Figure 7). Similarly, IL-6 levels decreased from 4.4–4.7 ng/mL in the untreated cells to 3.2–3.5 ng/mL in the IC_50_ treatment (Figure 7). A 25–30% decrease in IFN-γ levels was observed. A slightly increased TNF-α level was detected, which was thought to be due to a low-dose inflammatory stress response. These results indicate that OCT-D suppresses the inflammatory response in the RPMI-2650 line derived from nasal epithelial cells. ELISA analyses performed on HUVEC also yielded similar results. IL-6, IL-1β, TNF-α, and IFN-γ levels in HUVEC decreased significantly after OCT-D administration (Figure 7). IL-6 levels, in particular, decreased from 6.2–6.6 ng/mL in the untreated cells to 5.2–5.5 ng/mL at IC_50_ (Figure 7). Similar notable decreases were observed in TNF-α and IL-1β levels. The nearly 30% decrease in IFN-γ levels demonstrates the suppressive effect of OCT-D on the pro-inflammatory signaling pathway in endothelial cells. Overall, the ELISA data showed that OCT-D suppressed the inflammatory response by reducing cytokine levels in both cell lines. This modulation, particularly observed in endothelial-derived HUVEC, suggests that OCT-D may be a potential topical agent for the treatment of vascular inflammation and related diseases.

### 3.7. Effect of OCT-D on mRNA Expression Levels

**Figure 8:**
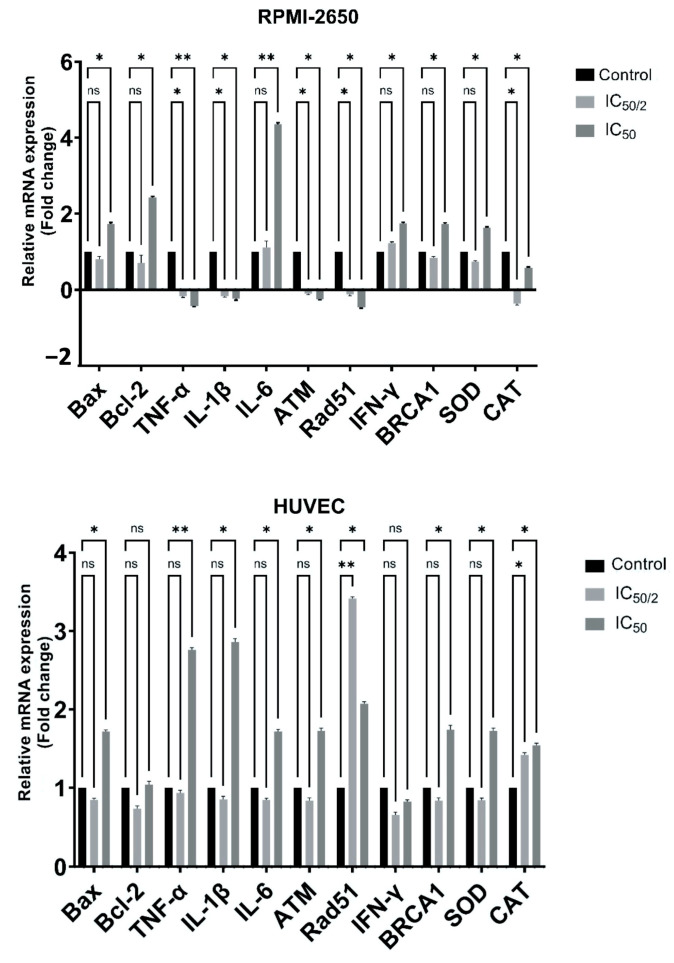
Evaluation of the effect of OCT-D on mRNA expression level in RPMI-2650 and HUVEC for 12 h [IC_50_: 50 percent inhibitory concentration (% 0.05 OCT-D), ns: no significance, * *p* < 0.005, ** *p* < 0.0001)].

OCT-D treatment led to marked changes in the mRNA expression of inflammatory cytokines in RPMI-2650 cells (Figure 8). TNF-α and IL-1β levels were statistically significantly decreased at ½ IC_50_ and IC_50_ concentrations, respectively (*p* < 0.005 and *p* < 0.0001). IL-6 expression showed a pronounced decrease (*p* < 0.0001) only at the IC_50_ concentration (Figure 8). Expression of the antioxidant enzymes *SOD* and *CAT* genes decreased at both OCT-D concentrations, and this effect was statistically significant, particularly in the IC_50_ group (*p* < 0.05) (Figure 8). Similar decreases were observed in the apoptosis-related *Bax* and *Bcl-2* genes. Expression of the DNA repair genes *ATM* and *Rad51* increased (*p* < 0.05) at the ½ IC_50_ concentration of OCT-D, while no marked change was detected in the BRCA1 gene (Figure 8). These findings suggest that OCT-D plays a role in suppressing inflammation and oxidative stress in RPMI-2650 cells.

OCT-D administration in HUVEC caused marked decreases (*p* < 0.005) in the mRNA levels of pro-inflammatory cytokines such as TNF-α, IL-1β, and IL-6 (Figure 8). IFN-γ expression, however, did not show a statistically notable change. Expression of the antioxidant enzymes *SOD* and *CAT* genes decreased significantly, particularly at the IC_50_ concentration (*p* < 0.05) (Figure 8). A decrease (*p* < 0.05) was observed in the apoptosis-associated *Bax* gene in the IC_50_ group, while no significant difference was detected in the *Bcl-2* gene (Figure 8). Expression of the DNA repair genes *ATM* and *BRCA1* decreased (*p* < 0.05) at the IC_50_ concentration of OCT-D (Figure 8). A significant decrease (*p* < 0.05) was noted in the *Rad51* gene in the ½ IC_50_ group. These results supported the anti-inflammatory and antioxidant effects of OCT-D in HUVEC (Figure 8).

## 4. Discussion

This study comprehensively evaluated the cytotoxic, genotoxic, apoptotic, oxidative, and inflammatory responses induced by OCT-D in RPMI-2650 nasal epithelial carcinoma and HUVEC endothelial cells. OCT-D exhibited dose- and time-dependent cytotoxicity, with markedly higher apoptotic sensitivity in HUVECs compared to RPMI-2650 cells. Genotoxicity assays demonstrated moderate but significant DNA damage and micronucleus formation in both cell lines, while oxidative and cytokine analyses revealed reduced ROS production and decreased pro-inflammatory cytokine levels following treatment. Gene expression profiling further supported these biochemical changes, showing differential regulation of apoptosis-, oxidative stress-, and inflammation-related genes. All the results demonstrated that OCT-D exhibited minimal cytotoxic and apoptotic effects in RPMI-2650 cells, but affected vascular structure by inducing apoptosis in endothelial cells.

The obtained findings determined the dose- and time-dependent cytotoxic effects of OCT-D used in WST-1 assays. Minimal loss of viability was observed in RPMI-2650 cells even at IC_50_ doses of OCT-D, demonstrating that RPMI-2650 cells are relatively resistant to OCT-D. In contrast, a considerable decrease in viability and a dramatic increase in the proportion of apoptotic cells were observed in HUVEC. Annexin V flow cytometry analyses revealed that OCT-D is a potent inducer of apoptosis in endothelial cells, with a total apoptotic cell rate of up to 80% in HUVEC. This finding is consistent with literature studies that specifically target peritumoral neovascularization via apoptosis in endothelial cells with low-dose anticancer agents [24]. The lack of a notable increase in apoptotic rates in RPMI-2650 cells suggests that nasal epithelial cells exhibit a different biological response to OCT-D and potentially exhibit a treatment-resistant phenotype.

In genotoxicity analyses, comet and micronucleus assay results showed that OCT-D caused a dose-dependent, moderate increase in DNA damage and micronuclei (MN) in both cell lines. This demonstrated that OCT-D may have partial genotoxic effects directly on DNA, and this effect was particularly pronounced in endothelial cells. However, the lack of significant changes in head DNA percentages suggests that OCT-D does not cause large-scale double-strand breaks in DNA. The literature has reported that the genotoxic effects of antiseptic agents are generally limited and primarily affect cell membrane and mitochondrial functions [25].

An important finding of the study is the increased apoptotic sensitivity and vascular toxicity potential of OCT-D in HUVEC. While this is a positive feature for targeting the vascular structure in the tumor microenvironment, systemic use requires caution due to side effects. Inducing apoptosis in endothelial cells may contribute to the inhibition of tumor angiogenesis but may increase the risk of toxicity in healthy vascular tissue [26,27,28]. While the induction of apoptosis in endothelial cells may suggest a potential anti-angiogenic effect, this interpretation remains speculative, as HUVECs are normal, non-tumorigenic endothelial cells that do not mimic the tumor vasculature [29]. Moreover, no angiogenesis markers or functional assays were performed in this study to suport this interpretation. Therefore, the present findings should be regarded as preliminary in vitro observations. Further confirmation of possible anti-angiogenic activity would require in vivo or tumor co-culture models incorporating angiogenesis-related endpoints, which will be pursued in future studies. Additionally, apoptotic rates remained below 1% at ½ IC_50_ and IC_50_ concentrations, whereas viability markedly decreased. This apparent discrepancy may reflect the timing of analysis, since apoptosis can occur transiently or be followed by secondary necrosis. Moreover, other forms of regulated cell death, including necrosis or autophagy, might also contribute to the overall cytotoxic response [30]. Although these mechanisms were not specifically examined here, their potential involvement is acknowledged as a limitation and will be explored in future studies.

Reactive oxygen species (ROS) analysis revealed distinct patterns between cell types and treatment conditions. In control groups, basal ROS levels were substantially higher in RPMI-2650 cells (88.15%) compared to HUVECs (7.26%), which likely reflects intrinsic differences in cellular metabolism and redox homeostasis. Tumor-derived epithelial cells generally exhibit enhanced oxidative metabolism and NADPH oxidase activity, resulting in elevated ROS production, whereas primary endothelial cells maintain tighter redox regulation to preserve cellular integrity [31]. Nevertheless, methodological factors such as probe sensitivity, cell density, and dye uptake efficiency may also contribute to this variation, representing a limitation of the current study.

Following treatment, RPMI-2650 cells exhibited a marked decrease in ROS levels after 12 h, which may be explained by rapid ROS scavenging, exhaustion of oxidative capacity, or selective loss of highly stressed cells no longer detectable at the time of measurement. The apparent reduction in ROS therefore likely reflects a late-stage oxidative collapse rather than a simple suppression of ROS generation [32]. Overall, these findings suggest that treatment-induced redox alterations differ between tumor and endothelial cell types, and future studies employing complementary time-course and probe-based assays are warranted to confirm and further characterize these redox dynamics [33].

In the evaluation of inflammatory responses, a significant decrease was observed in IL-1β, IFN-γ, TNF-α, and IL-6 levels measured by ELISA after OCT-D administration. While OCT-D significantly reduced pro-inflammatory cytokine levels in vitro, these findings should be interpreted as indicative of general anti-inflammatory potential rather than direct evidence of carcinoma-related inflammation control. The possible link between inflammation and nasal carcinoma remains speculative and would require further validation through in vivo or clinical studies. Given that inflammation plays an important role in the pathogenesis of nasal septum squamous carcinoma, these findings may support the combination use of OCT-D in treatment strategies [34]. Furthermore, studies have reported that OCT-D inhibits the secretion of inflammatory cytokines (IL-8, IL-33, IL-10) and accelerates wound healing [35]. These properties increase the potential of OCT-D as a topical treatment in pathologies where inflammation plays a role.

Additionally, the observed decreases in cytokine levels following treatment should be interpreted with caution. As cell viability was substantially reduced under these conditions, part of the decrease may be attributable to reduced viable cell number rather than a direct suppression of cytokine synthesis. In this study, cytokine measurements were performed on culture supernatants without normalization to total protein or cell count, which represents a methodological limitation. Nevertheless, the consistent pattern of cytokine reduction across independent experiments suggests a possible contribution of anti-inflammatory signaling alongside cell death-related effects [36]. To better clarify this relationship, future studies will include normalization to cell number or total protein content and time-resolved cytokine analyses to confirm these findings.

Molecular RT-PCR analyses revealed notable changes in the expression of the apoptotic genes *Bax* and *Bcl-2* after OCT-D treatment. In RPMI-2650 cells, an increase in *Bax* expression accompanied by a decrease in *Bcl-2* supports the activation of apoptosis at the transcriptional level. In contrast, HUVEC did not show similar changes, suggesting that apoptosis in these cells is likely regulated at the post-transcriptional or protein level rather than through mRNA expression. Furthermore, the observed alterations in the antioxidant enzyme genes *SOD* and *CAT* indicate an adaptive cellular response to oxidative stress. The literature supports that oxidative stress contributes to tumor progression in squamous cell carcinomas and that antioxidant defense mechanisms can influence therapeutic response [29,37,38]. Annexin V analysis revealed a marked increase in apoptosis in HUVECs, whereas *Bax* and *Bcl-2* mRNA expression showed little or no change. This mismatch suggests that apoptotic regulation occurred mainly at the post-transcriptional or post-translational level rather than through transcriptional control. Mitochondrial membrane depolarization, caspase activation, or protein-level modulation of *Bcl-2* family members may account for the observed effects [39]. In addition, temporal differences between gene expression and apoptosis detection may have contributed to this divergence. Overall, these results indicate that the treatment influences redox balance and cell-death signaling through multiple interconnected mechanisms [40]. Future mechanistic studies incorporating time-course analyses, protein-level validation, and specific pathway inhibitors will further clarify these regulatory interactions. This issue will be further addressed in upcoming mechanistic studies.

Although both SOD and CAT expression were found to decrease following treatment, intracellular ROS levels also declined. This apparent discrepancy may be explained by the direct ROS-scavenging or radical-quenching properties of the treatment compound, leading to a reduction in oxidative stress independent of endogenous antioxidant enzyme induction [41]. Once ROS levels drop below a certain threshold, the expression of antioxidant genes such as SOD and CAT may be transcriptionally downregulated through feedback regulation mechanisms. Alternatively, the observed decrease could reflect reduced cellular activity due to cytotoxic effects [42]. Future studies including direct ROS scavenging assays and kinetic analyses of antioxidant gene expression will help to further elucidate these redox dynamics. This issue will be further addressed in upcoming mechanistic studies.

While the literature on the effects of OCT-D on nasal treatment and disease course in clinical cases is limited, existing studies provide significant evidence that OCT-D may offer distinct benefits with local applications. Intranasal OCT-D administration, in particular, has been shown to be effective in reducing *S. aureus* and MRSA carriage and reducing the risk of post-surgical infection [43,44]. Furthermore, the findings in our study support OCT-D’s capacity to inhibit tumor cell proliferation and reduce inflammation in vitro, suggesting a potential role that warrants further investigation as an adjuvant treatment after surgery. Controlling local post-surgical inflammation and preventing microbial contamination may positively impact patient recovery and reduce the risk of recurrence [45].

One limitation of this study is that only 25 nuclei per treatment group were analyzed in the comet assay, which may reduce the statistical power. Future studies should include a larger number of comets and biological replicates to enhance the robustness of genotoxicity assessments.

## 5. Conclusions

In conclusion, this study demonstrated that OCT-D exhibited minimal cytotoxic and apoptotic effects on RPMI-2650 cells, but induced significant apoptosis in endothelial cells, affecting vascular structure. While the genotoxic effect observed was limited and may be within tolerable limits for nasal mucosa applications, these findings are based solely on in vitro data. These findings provide significant evidence that OCT-D can be used as a potential adjunctive agent in nasal therapies, and our data should be supported by preclinical and clinical studies.

## Data Availability

The original contributions presented in this study are included in the article. Further inquiries can be directed to the corresponding author.

## References

[1] Bührer C., Bahr S., Siebert J., Wettstein R., Geffers C., Obladen M. (2002). Use of 2% 2-phenoxyethanol and 0.1% octenidine as antiseptic in premature newborn infants of 23–26 weeks gestation. J. Hosp. Infect..

[2] Alvarez-Marin R., Aires-de-Sousa M., Nordmann P., Kieffer N., Poirel L. (2017). Antimicrobial Activity of Octenidine against Multidrug-Resistant Gram-Negative Pathogens. Eur. J. Clin. Microbiol. Infect. Dis..

[3] Heckel M., Geißdörfer W., Herbst F.A., Stiel S., Ostgathe C., Bogdan C. (2017). Nasal Carriage of Methicillin-Resistant *Staphylococcus aureus* (MRSA) at a Palliative Care Unit: A Prospective Single Service Analysis. PLoS ONE.

[4] Allport J., Choudhury R., Bruce-Wootton P., Reed M., Tate D., Malviya A. (2022). Efficacy of Mupirocin, Neomycin and Octenidine for Nasal *Staphylococcus aureus* Decolonisation: A Retrospective Cohort Study. Antimicrob. Resist. Infect. Control..

[5] Sipahi O.R. (2008). Economics of Antibiotic Resistance. Expert Rev. Anti-Infect. Ther..

[6] Malanovic N., Ön A., Pabst G., Zellner A., Lohner K. (2020). Octenidine: Novel Insights into the Detailed Killing Mechanism of Gram-Negative Bacteria at a Cellular and Molecular Level. Int. J. Antimicrob. Agents.

[7] Mert G., Kilic A., Bedir O., Basustaoglu A.C. (2011). Clinical Significance and Staphylococcal Cassette Chromosome *mec* (SCCmec) Characterization of Coagulase-Negative Staphylococci Isolated from Blood Cultures. Turk. J. Med. Sci..

[8] Seah C., Alexander D.C., Louie L., Simor A., Low D.E., Longtin J., Melano R.G. (2012). MupB, a New High-Level Mupirocin Resistance Mechanism in *Staphylococcus aureus*. Antimicrob. Agents Chemother..

[9] Telchik C., Jinadatha C., Cadnum J., Donskey C., Chatterjee P., Dhar S., Kaye K., Yakubik T., Hwang M., Grimbergen A. (2024). Assessing Chlorhexidine Resistance in MRSA Isolates from Hospitals in Cleveland, OH and Detroit, MI. Antimicrob. Steward. Healthc. Epidemiol..

[10] Hübner N.O., Siebert J., Kramer A. (2010). Octenidine Dihydrochloride, a Modern Antiseptic for Skin, Mucous Membranes and Wounds. Skin Pharmacol. Physiol..

[11] Schmidt J., Zyba V., Jung K., Rinke S., Haak R., Mausberg R.F., Ziebolz D. (2016). Cytotoxic Effects of Octenidine Mouth Rinse on Human Fibroblasts and Epithelial Cells—An In Vitro Study. Drug Chem. Toxicol..

[12] Moorhead P.S. (1965). Human tumor cell line with a quasi-diploid karyotype (RPMI 2650). Exp. Cell Res..

[13] Sibinovska N., Žakelj S., Kristan K. (2019). Suitability of RPMI 2650 Cell Models for Nasal Drug Permeability Prediction. Eur. J. Pharm. Biopharm..

[14] Mercier C., Perek N., Delavenne X. (2018). Is RPMI 2650 a Suitable In Vitro Nasal Model for Drug Transport Studies?. Eur. J. Drug Metab. Pharmacokinet..

[15] Tice R.R., Agurell E., Anderson D., Burlinson B., Hartmann A., Kobayashi H., Miyamae Y., Rojas E., Ryu J.-C., Sasaki Y.F. (2000). Single Cell Gel/Comet Assay: Guidelines for In Vitro and In Vivo Genetic Toxicology Testing. Environ. Mol. Mutagen..

[16] Hayashi M. (2016). The Micronucleus Test—Most Widely Used in Vivo Genotoxicity Test. Genes Environ..

[17] Liu K., Liu P.C., Liu R., Wu X. (2015). Dual AO/EB Staining to Detect Apoptosis in Osteosarcoma Cells Compared with Flow Cytometry. Med. Sci. Monit. Basic Res..

[18] Nath S., Devi G.R. (2016). Three-Dimensional Culture Systems in Cancer Research: Focus on Tumor Spheroid Model. Pharmacol. Ther..

[19] Kwak H.H., Park J.H., Kim H.S., Lee H.M., Kim S.D., Mun S.J., Cho K.S. (2025). Inflammatory Effects of Particulate Matter Exposure on the Nasal and Paranasal Sinus Mucosa in Rats. Int. J. Mol. Sci..

[20] Mitupatum T., Aree K., Kittisenachai S., Roytrakul S., Puthong S., Kangsadalampai S., Rojpibulstit P. (2016). mRNA Expression of Bax, Bcl-2, p53, Cathepsin B, Caspase-3 and Caspase-9 in the HepG2 Cell Line Following Induction by a Novel Monoclonal Ab Hep88 mAb: Cross-Talk for Paraptosis and Apoptosis. Asian Pac. J. Cancer Prev..

[21] Liu G., Wang T., Wang T., Song J., Zhou Z. (2013). Effects of Apoptosis-Related Proteins Caspase-3, Bax and Bcl-2 on Cerebral Ischemia Rats. Biomed. Rep..

[22] Nakano T., Takahashi T., Yamamoto C., Yoshida E., Kaji T., Fujiwara Y. (2021). Arsenite Inhibits Tissue-Type Plasminogen Activator Synthesis through NRF2 Activation in Cultured Human Vascular Endothelial EA.hy926 Cells. Int. J. Mol. Sci..

[23] Møller P., Azqueta A., Sanz-Serrano J., Bakuradze T., Richling E., Eyluel Bankoglu E., Stopper H., Claudino Bastos V., Langie S.A.S., Jensen A. (2023). Visual comet scoring revisited: A guide to scoring comet assay slides and obtaining reliable results. Mutagenesis.

[24] Chum J.D., Lim D.J.Z., Sheriff S.O., Pulikkotil S.J., Suresh A., Davamani F. (2019). In Vitro Evaluation of Octenidine as an Antimicrobial Agent against *Staphylococcus epidermidis* in Disinfecting the Root Canal System. Restor. Dent. Endod..

[25] Shahneh F.Z., Baradaran B., Orangi M., Zamani F. (2013). In Vitro Cytotoxic Activity of Four Plants Used in Persian Traditional Medicine. Adv. Pharm. Bull..

[26] Ozkiriş M., Akbulut S., Aydın E., Unver S. (2006). Squamous Cell Carcinoma Originating from the Nasal Septum: A Case Report. J. Ear Nose Throat.

[27] Seiser S., Janker L., Zila N., Mildner M., Rakita A., Matiasek J., Bileck A., Gerner C., Paulitschke V., Elbe-Bürger A. (2021). Octenidine-Based Hydrogel Shows Anti-Inflammatory and Protease-Inhibitory Capacities in Wounded Human Skin. Sci. Rep..

[28] Cory S., Huang D.C.S., Adams J.M. (2003). The Bcl-2 Family: Roles in Cell Survival and Oncogenesis. Oncogene.

[29] Viallard C., Larrivée B. (2017). Tumor Angiogenesis and Vascular Normalization: Alternative Therapeutic Targets. Angiogenesis.

[30] Newton K., Strasser A., Kayagaki N., Dixit V.M. (2024). Cell Death. Cell.

[31] Chen D., Guo Z., Yao L., Sun Y., Dian Y., Zhao D., Ke Y., Zeng F., Zhang C., Deng G. (2025). Targeting Oxidative Stress-Mediated Regulated Cell Death as a Vulnerability in Cancer. Redox Biol..

[32] Zeng Z., Centner C., Gollhofer A., König D. (2021). Effects of Dietary Strategies on Exercise-Induced Oxidative Stress: A Narrative Review of Human Studies. Antioxidants.

[33] Song P., Zou M.H. (2014). Redox Regulation of Endothelial Cell Fate. Cell Mol. Life Sci..

[34] Hong Y., Boiti A., Vallone D., Foulkes N.S. (2024). Reactive Oxygen Species Signaling and Oxidative Stress: Transcriptional Regulation and Evolution. Antioxidants.

[35] Rius-Pérez S., Pérez S., Toledano M.B., Sastre J. (2023). Mitochondrial Reactive Oxygen Species and Lytic Programmed Cell Death in Acute Inflammation. Antioxid. Redox Signal..

[36] Wu H., Cui J., Huang J., Feng Y., Zhao J., Zhu Y., Deng X., Li X., Zhang W., Wang C. (2025). Cell Death Signaling and Immune Regulation: New Perspectives on Targeted Therapy for Sepsis. Cell Mol. Biol. Lett..

[37] Gao F., Xu T., Zang F., Luo Y., Pan D. (2024). Cardiotoxicity of Anticancer Drugs: Molecular Mechanisms, Clinical Management and Innovative Treatment. Drug Des. Dev. Ther..

[38] Liu Z.L., Chen H.H., Zheng L.L., Sun L.P., Shi L. (2023). Angiogenic Signaling Pathways and Anti-Angiogenic Therapy for Cancer. Signal Transduct. Target. Ther..

[39] Vogler M., Braun Y., Smith V.M., Westhoff M.-A., Pereira R.S., Pieper N.M., Anders M., Callens M., Vervliet T., Abbas M. (2025). The BCL2 family: From apoptosis mechanisms to new advances in targeted therapy. Signal Transduct. Target. Ther..

[40] Li B., Ming H., Qin S., Nice E.C., Dong J., Du Z., Huang C. (2025). Redox Regulation: Mechanisms, Biology and Therapeutic Targets in Diseases. Signal Transduct. Target. Ther..

[41] Liu J., Han X., Zhang T., Tian K., Li Z., Luo F. (2023). Reactive Oxygen Species (ROS) Scavenging Biomaterials for Anti-Inflammatory Diseases: From Mechanism to Therapy. J. Hematol. Oncol..

[42] Maître B., Jornot L., Junod A.F. (1993). Effects of Inhibition of Catalase and Superoxide Dismutase Activity on Antioxidant Enzyme mRNA Levels. Am. J. Physiol..

[43] Köck R., Denkel L., Feßler A.T., Eicker R., Mellmann A., Schwarz S., Geffers C., Hübner N.-O., Leistner R. (2023). Clinical Evidence for the Use of Octenidine Dihydrochloride to Prevent Healthcare-Associated Infections and Decrease *Staphylococcus aureus* Carriage or Transmission—A Review. Pathogens.

[44] Unterfrauner I., Bragatto-Hess N., Studhalter T., Farshad M., Uçkay I. (2024). General Skin and Nasal Decolonization with Octenisan^®^ Set before and after Elective Orthopedic Surgery in Selected Patients at Elevated Risk for Revision Surgery and Surgical Site Infections—A Single-Center, Unblinded, Superiority, Randomized Controlled Trial (BALGDEC Trial). Trials.

[45] Haesler E. (2020). Evidence Summary: Octenidine for Chronic Wounds. Wound Pract. Res..

